# Effect of Different Adjuvants on Immune Responses Elicited by Protein-Based Subunit Vaccines against SARS-CoV-2 and Its Delta Variant

**DOI:** 10.3390/v14030501

**Published:** 2022-02-28

**Authors:** Naru Zhang, Qianting Ji, Zezhong Liu, Kaiming Tang, Yubin Xie, Kangchen Li, Jie Zhou, Sisi Li, Haotian Shang, Zecan Shi, Tianyu Zheng, Jiawei Yao, Lu Lu, Shuofeng Yuan, Shibo Jiang

**Affiliations:** 1 School of Medicine and Waikato Joint Institute, Zhejiang University City College, Hangzhou 310015, China; zhangnr@zucc.edu.cn (N.Z.); 31904194@stu.zucc.edu.cn (Q.J.); 31904208@stu.zucc.edu.cn (K.L.); lisisi@zucc.edu.cn (S.L.); 31904028@stu.zucc.edu.cn (H.S.); 31904213@stu.zucc.edu.cn (Z.S.); 32011221@stu.zucc.edu.cn (T.Z.); 32004146@stu.zucc.edu.cn (J.Y.); 2 Key Laboratory of Medical Molecular Virology (MOE/NHC/CAMS), Shanghai Frontiers Science Center of Pathogenic Microbes and Infection, School of Basic Medical Sciences, Shanghai Institute of Infectious Disease and Biosecurity, Fudan University, Shanghai 200032, China; zzliu17@fudan.edu.cn (Z.L.); 21111010093@m.fudan.edu.cn (J.Z.); 3 Department of Microbiology, Li Ka Shing Faculty of Medicine, The University of Hong Kong, Hong Kong, China; kmtang21@connect.hku.hk (K.T.); xieyubin@hku.hk (Y.X.)

**Keywords:** COVID-19, SARS-CoV-2, variants, aluminum, nanoparticle manganese, MF59, subunit vaccines

## Abstract

The global pandemic of coronavirus disease 2019 (COVID-19) caused by severe acute respiratory syndrome coronavirus 2 (SARS-CoV-2) has become more serious because of the continuous emergence of variants of concern (VOC), thus calling for the development of broad-spectrum vaccines with greater efficacy. Adjuvants play important roles in enhancing the immunogenicity of protein-based subunit vaccines. In this study, we compared the effect of three adjuvants, including aluminum, nanoparticle manganese and MF59, on the immunogenicity of three protein-based COVID-19 vaccine candidates, including RBD-Fc, RBD and S-trimer. We found that the nanoparticle manganese adjuvant elicited the highest titers of SARS-CoV-2 RBD-specific IgG, IgG1 and IgG2a, as well as neutralizing antibodies against infection by pseudotyped SARS-CoV-2 and its Delta variant. What is more, the nanoparticle manganese adjuvant effectively reduced the viral load of the authentic SARS-CoV-2 and Delta variant in the cell culture supernatants. These results suggest that nanoparticle manganese, known to facilitate cGAS-STING activation, is an optimal adjuvant for protein-based COVID-19 subunit vaccines.

## 1. Introduction

Coronavirus disease 2019 (COVID-19), which is caused by the pathogen of severe acute respiratory syndrome coronavirus 2 (SARS-CoV-2), has become increasingly serious as a result of the continuous emergence of variants of concern (VOC), including B.1.1.7 (Alpha) [1], B.1.351 (Beta) [2], P.1 (Gamma) [3], B.1.617.2 (Delta) [4] and B.1.1.529 (Omicron) [5]. As of January 21, 2022, the virus had caused 340,543,962 confirmed cases globally and 5,570,163 deaths [6]. As of July 2021, more than 292 COVID-19 vaccine candidates had been developed, with 184 in human preclinical trials [7]. By 18 January 2022, a total of 9,571,502,663 vaccine doses had been administered [6]. However, scientific consensus is growing that current vaccines show relatively low neutralizing immunogenicity, no broad-spectrum efficacy, and little lasting protective immunity [8]. To address this grim outlook, the development of more effective, long-lasting and broad-spectrum vaccines is essential. Adjuvants are considered critical components for both subunit vaccines and certain inactivated vaccines [9,10]. Multimeric display of antigens in combination with a powerful adjuvant could enhance the longevity and potency of induced immune responses in hosts. Thus, adjuvanted vaccines could induce more effective, broad-spectrum and durable protective immunity against SARS-CoV-2 and its emerging variants.

Recently, we found a new stimulator of interferon gene (STING) agonist and named it CF501. The CF501-adjuvanted RBD-Fc vaccine (CF501/RBD-Fc) was shown to elicit potent neutralizing antibodies (nAbs) against SARS-CoV-2 and its 9 variants and 41 S-mutants, SARS-CoV and bat SARSr-CoVs in mice, rabbits and rhesus macaques [11]. Nanoparticle manganese adjuvant, also a STING agonist, was shown to facilitate antigen presentation, virus-specific memory T cell development and host-adaptive immunity [12,13]. MF59 was licensed for human use in Europe. As shown in our previously published paper, it promoted immunogenicity of Middle East respiratory syndrome (MERS) vaccines with an excellent safety profile [14]. Aluminum hydroxide (or alum), a licensed adjuvant in the United States and most developing countries, induced strong immune responses, but it did not efficiently enhance T helper 1 (Th1) cellular immune responses [9]. In this study, we evaluated the effects of nanoparticle manganese, MF59 and alum adjuvants on the induction of host immune responses by using RBD-Fc, RBD and S-trimer of SARS-CoV-2 as antigens in BALB/c mice. RBD-specific IgG and specific neutralizing antibodies of SARS-CoV-2 and its Delta variant elicited in mice were systematically compared. We found the nanoparticle manganese adjuvant to be the most potent in enhancing immunogenicity or immune responses of SARS-CoV-2 protein-based subunit vaccines.

## 2. Materials and Methods

### 2.1. Ethics Statement

Six-week-old specific-pathogen-free (SPF) female BALB/c mice, which are frequently used to evaluate the efficacy of vaccine candidates, were purchased from Beijing Vital River Laboratory Animal Technology Co., Ltd. All mice were in good health. Animal studies were carried out in strict accordance with recommendations of the Institutional Animal Care and Use Committee (IACUC), Institute of Laboratory Animal Science, Chinese Academy of Medical Sciences (DW21002), and the animal protocol was approved by the Committee on the Ethics of Animal Experiments of School of Basic Medical Sciences, Fudan University (permission code: 20200201-002; permission date: 1 February 2020).

### 2.2. Cell Lines, Proteins, Adjuvants and Viruses

HEK293T, Huh-7, Vero-E6 and VeroE6-TMPRSS2 cells were obtained from the American Type Culture Collection (ATCC), and cells were cultured and propagated in Dulbecco’s Modified Eagle’s Medium (DMEM) (Meilun, Dalian, China) supplemented with 10% fetal bovine serum (FBS) (Gibco, New York, NY, USA), streptomycin (100 mg/mL) (Thermo Fisher Scientific, Shanghai, China) and penicillin (100 U/mL) (Thermo Fisher Scientific, Shanghai, China). RBD-Fc, RBD and S-trimer proteins were purchased from Kactusbio (Shanghai, China). Alum adjuvant was purchased from Thermo Scientific (Shanghai, China). The manganese adjuvant was kindly provided by Dr. Zhengfan Jiang of the School of Life Sciences, Peking University. MF59 adjuvant was prepared as previously described [14]. Briefly, 4.3% squalene (Sigma, St Louis, MO, USA) was mixed with 5% Span 85 (Sigma, St Louis, MO, USA) and 5% Tween 80 (Macklin, Shanghai, China) in 10 nM of sodium citrate (pH 6.5), followed by sonication for 5 min at 2000 W. The solution was filtered through a 0.22 µm filter membrane (Millipore, Shanghai, China) and stored at 4 °C before use.

The authentic SARS-CoV-2 HKU-001a strain (GenBank accession number: MT230904) and SARS-CoV-2 B.1.617.2/Delta (GISAID: EPI_ISL_3221329) strain were isolated from the respiratory tract specimens of laboratory-confirmed COVID-19 patients in Hong Kong. All experiments involving authentic SARS-CoV-2 followed the approved standard operating procedures of the biosafety level 3 facility at the University of Hong Kong.

### 2.3. Animal Vaccination and Sample Collection

**Figure 1:**
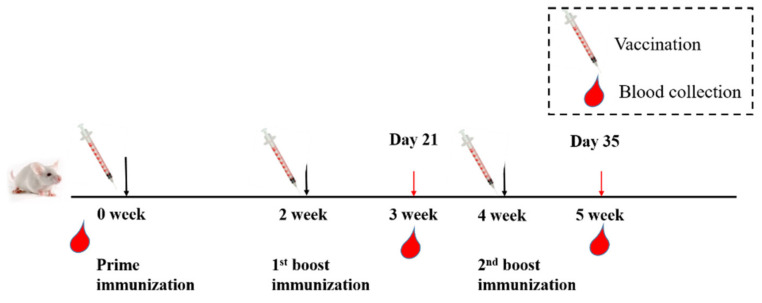
Schematic diagram of immunization strategy and sample collection. Twelve groups of mice (five mice/group) were intramuscularly immunized with RBD-Fc, RBD and S-trimer plus PBS, alum, MF59 or nanoparticle manganese adjuvant, respectively, three times at two-week intervals. Sera were collected one week after each immunization for antibody detection and viral load reduction assay.

Sixty BALB/c mice were randomly assigned to 12 groups, and each group had 5 mice. All mice were intramuscularly immunized as follows. Groups 1 to 4 were immunized with 5 µg of RBD-Fc antigen (equal to 0.99 µM) formulated with an equal volume of PBS, alum adjuvant, MF59 or 100 µg manganese adjuvant, respectively. Groups 5 to 8 were immunized with 5 µg of RBD antigen (equal to 1.85 µM) formulated with an equal volume of PBS, alum adjuvant, MF59 or 100 µg manganese adjuvant, respectively. Groups 8 to 12 were immunized with 5 µg of S-trimer antigen (equal to 0.37 µM) formulated with an equal volume of PBS, alum adjuvant, MF59 or 100 µg manganese adjuvant, respectively. The immunization strategy is shown in Figure 1. All mice were immunized three times at 2-week intervals. Sera were isolated from collected blood one week after each immunization to detect RBD-specific antibodies and neutralizing antibodies (nAbs) against both pseudotyped and authentic SARS-CoV-2, as well as its Delta variant.

### 2.4. ELISA

Enzyme-linked immunosorbent assay (ELISA) was used to quantify the SARS-CoV-2 RBD-specific IgG, IgG1 and IgG2a titers in serum based on a previous report [15], with minor modification. Briefly, ELISA plates were pre-coated with 2 µg/mL RBD of wildtype SARS-CoV-2 overnight at 4 °C. The coated plates were washed with phosphate-buffered saline (PBS) containing 0.05% Tween (PBS-T) and blocked with PBS containing 2.5% bovine serum albumin for 2 h at 37 °C. Mouse sera were serially diluted in PBS-T, followed by incubation of plates at 37 °C for 1 h. After four washes with PBS-T, HRP-conjugated goat anti-mouse IgG (Beyotime, Shanghai, China), goat anti-mouse IgG1 (abcam, Shanghai, China) or goat anti-mouse IgG2a (abcam, Shanghai, China) was applied to plates and incubated at 37 °C for 1 h. After the plates were washed thoroughly, 3, 3′, 5, 5′-Tetramethylbenzidine (TMB) (Beyotime, Shanghai, China) was added into the plates to visualize the reaction. Finally, 1 N H_2_SO_4_ was added into the plates to stop the reaction, and the absorbance value was collected at 450 nm using a Tecan Infinite M200PRO microplate reader (Männedorf, Switzerland). The endpoint titers were expressed as the highest reciprocal serum dilution exhibiting an absorbance of 450 nm > 2.1-fold over the background values.

### 2.5. Pseudovirus Production

Generation of both pseudotyped wildtype SARS-CoV-2 and its Delta variant was performed as previously described [11,16,17,18]. Briefly, the backbone plasmid of pNL4-3.Luc.R-E- was co-transfected with the plasmid of pcDNA3.1-SARS-CoV-2-S (D614G)/pcDNA3.1-SARS-CoV-2-S (Delta) into HEK-293T cells by using VigoFect transfection reagent (Vigorous Biotechnology, Beijing, China). Cell culture supernatant containing the pseudoviruses was harvested 60 h post-transfection. The pseudotyped viruses were stored at −80 °C until use.

### 2.6. Pseudovirus Neutralization Assay

Detection of nAbs against pseudotyped SARS-CoV-2 and its Delta variant was conducted as previously described [11,19]. Briefly, the mouse sera from all 12 groups were inactivated at 56 °C for 30 min. Huh-7 cells were seeded at a density of 1 × 10^4^/well in 96-well cell culture plates. Serially diluted mouse sera were incubated with the pseudotyped viruses for 30 min. Then, the mixture of mouse sera and pseudoviruses was transferred to 96-well cell culture plates. After 12 h, the cell culture supernatant was discarded, and cell culturing continued with fresh DMEM containing 2% FBS for 48 h. Promega 1X lysis buffer was added into the wells for 30 min, the lysate was transferred into 96-well half-area white plates, and the luciferase activity was detected by a Firefly luciferase assay kit (Promega, Madison, WI, USA). Luciferase values were measured using a Tecan Infinite M200PRO microplate reader (Männedorf, Switzerland). Neutralization antibody titers (NT_50_) were defined as the serum dilutions that could reduce 50% relative luminescence units compared to virus control wells.

### 2.7. Viral Load Reduction Assay

Viral load reduction assay was performed on VeroE6-TMPRSS2 cells as described previously [20], with minor modifications. Serially diluted serum was pre-incubated with 1000 PFU of the virus for 1 h before infecting the cells in 96-well cell culture plates for another 1 h. Afterwards, the infectious inoculum was aspirated, washed and replaced with fresh medium containing the serially diluted serum. Forty-eight hours later, supernatant samples from the infected cells were harvested for qRT-PCR analysis of virus replication. Briefly, 100 µL of viral supernatant was lysed with 400 µL of AVL buffer and then extracted for total RNA with a QIAamp viral RNA mini kit (Qiagen, Hilden, Germany). Real-time one-step qRT-PCR was used for quantitation of viral load using a QuantiNova Probe RT-PCR kit (Qiagen) with a LightCycler 480 real-time PCR system (Roche). Each 20 µL reaction mixture contained 10 µL of 2× QuantiNova Probe RT-PCR master mix, 1.2 µL of RNase-free water, 0.2 µL of QuantiNova Probe RT-Mix, 1.6 µL each of 10 µM forward and reverse primer, 0.4 µL of 10 µM probe, and 5 µL of extracted RNA as the template. Reactions were incubated at 45 °C for 10 min for reverse transcription and 95 °C for 5 min for denaturation, followed by 45 cycles of 95 °C for 5 s and 55 °C for 30 s. Signal detection and measurement were performed in each cycle after the annealing step. The cycling profile ended with a cooling step at 40 °C for 30 s. The primers and probe sequences were against the RNA-dependent RNA polymerase/Helicase (RdRP/Hel) gene region of SARS-CoV-2: forward primer: 5′ CGCATACAGTCTTRCAGGCT-3′; reverse primer: 5′-GTGTGATGTTGAWATGACATGGTC-3′; specific probe: 5′-FAM TTAAGATGTGGTGCTTGCATACGTAGAC-IABkFQ-3.

### 2.8. Statistical Analysis

All statistical analyses were performed with GraphPad Prism 8 software. Statistical significance was calculated by two-way ANOVA. *p* values less than 0.05 were considered statistically significant.

## 3. Results

### 3.1. Manganese-Adjuvanted RBD-Fc Protein Elicited the Highest Titers of IgG Antibody in Immunized Mouse Sera

**Figure 2:**
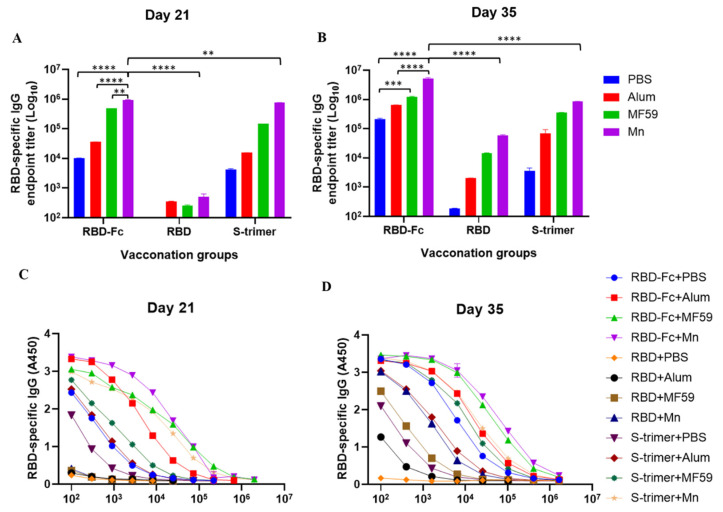
RBD-specific IgG detection in immunized mouse sera. The IgG in immunized mouse sera were detected with ELISA using the RBD protein to coat the plates. (**A**) RBD-specific IgG endpoint titer on day 21 in all groups of immunized mice. (**B**) RBD-specific IgG endpoint titer on day 35 in all groups of immunized mice. (**C**) The curve of RBD-specific IgG in immunized mice on day 21. (**D**) The curve of RBD-specific IgG in immunized mice on day 35. Geometric mean was calculated for each set of data, as shown and compared. Statistical significance was determined by two-way ANOVA and is indicated as follows: ** *p* < 0.01, *** *p* < 0.001, **** *p* < 0.0001. Error bars represent SD.

Mice were intramuscularly vaccinated with RBD-Fc, RBD or S-trimer plus alum, MF59 or nanoparticle manganese adjuvant three times at two-week intervals. RBD-Fc, RBD or S-trimer plus an equal volume of PBS were used as internal controls, and the sera were collected one week after each vaccination. The endpoint titers of IgG were detected with ELISA using RBD protein to coat the plates because the vaccinated mice with RBD-Fc antigen could produce anti-human Fc antibodies [15]. Based on sera collected from mice on day 21 (one week after the second vaccination), as shown in Figure 2A, a low level of immune response was observed in mice immunized with RBD. However, a slightly elevated antibody response was induced by RBD plus alum and MF59 adjuvants. The level of response was significantly increased in the group adjuvanted with nanoparticle manganese. A relatively higher level of immune response was observed in mice immunized with S-trimer, whereas, again, a slightly elevated antibody response was induced by S-trimer plus the three adjuvants. Compared with RBD and S-trimer, mice immunized with RBD-Fc exhibited a significantly higher level of immune response. Particularly elevated antibody responses were shown in the RBD-Fc groups adjuvanted with alum and MF59 adjuvants, respectively. Following the above trend, the RBD-Fc groups adjuvanted with nanoparticle manganese induced a significantly higher level when compared to the groups adjuvanted with alum and MF59. As shown in Figure 2B, the response in all groups reached a higher level on day 35 (one week after the third vaccination). Among the three adjuvants, nanoparticle manganese appeared to be the most potent in promoting RBD-specific antibody responses. We also tested the sera collected on days 21 and 35 for their capacities to bind to RBD protein. As shown in Figure 2C,D, the addition of adjuvants to RBD-Fc, RBD or S-trimer proteins improved their ability to bind to RBD protein, with, as noted above, RBD-Fc plus nanoparticle manganese being the most potent, followed by RBD-Fc plus MF59 as the second most potent, S-trimer plus nanoparticle manganese as the third most potent and RBD-Fc plus alum as the fourth most potent.

### 3.2. Manganese-Adjuvanted RBD-Fc Protein Induced the Highest Titers of IgG1 and IgG2a Subtype Antibodies in Immunized Mice

**Figure 3:**
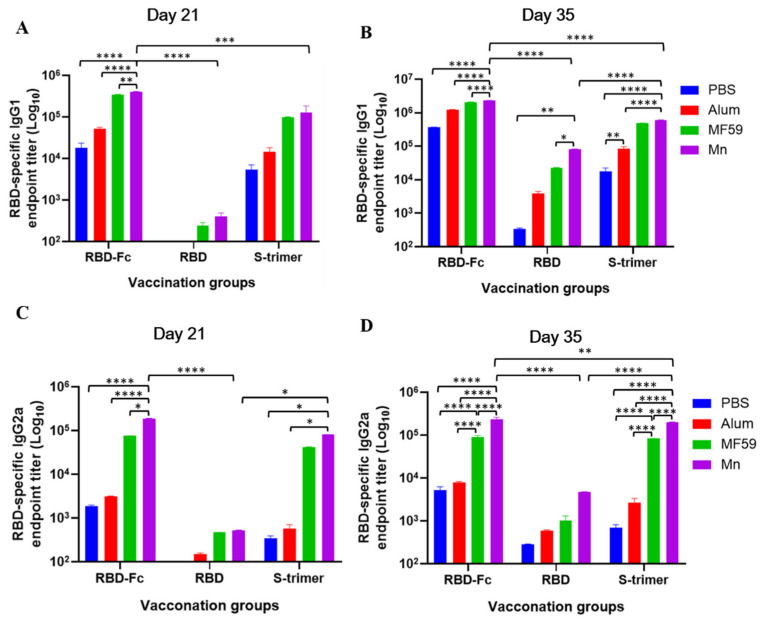
Titers of serum IgG1 and IgG2a antibodies to RBD protein in immunized mouse sera. RBD-specific IgG1 endpoint titer on day 21 (**A**) and on day 35 (**B**) in vaccinated mouse sera. RBD-specific IgG2a endpoint titer on day 21 (**C**) and on day 35 (**D**) in vaccinated mouse sera. Statistical significance was determined by two-way ANOVA and is indicated as follows: * *p* < 0.05, ** *p* < 0.01, *** *p* < 0.001, **** *p* < 0.0001. Error bars represent SD.

The endpoint titers of IgG1 and IgG2a in immunized mouse sera on days 21 and 35 were detected with ELISA. RBD protein was also used to coat the plates. As shown in Figure 3A,C, RBD protein without adjuvant induced nearly a background level of IgG1 and IgG2a antibody responses, with slightly elevated levels in adjuvanted groups on day 21. However, compared with RBD and S-trimer, RBD-Fc induced a significantly higher level of IgG1 and IgG2a, with nanoparticle manganese again showing the most potency. As shown in Figure 3B,D, both IgG1 and IgG2a in all groups reached a higher level on day 35. Nanoparticle-manganese-adjuvanted RBD-Fc induced a significantly higher level of IgG1 immune responses compared with RBD-Fc alone, RBD-Fc plus alum, and RBD-Fc plus MF59, with *p* < 0.0001 (Figure 3B). Nanoparticle-manganese-adjuvanted RBD-Fc also induced a significantly higher level of IgG2a immune responses compared with RBD-Fc alone, RBD-Fc plus alum, and RBD-Fc plus MF59, with *p* < 0.0001 (Figure 3D). When adjuvanted with the same nanoparticle manganese, RBD-Fc still induced a significantly higher titer of IgG1 and IgG2a than that of either RBD- or S-trimer-vaccinated groups. All groups showed the Th1 and Th2 balanced antibody response but with slightly Th2-biased cellular immune responses.

### 3.3. Manganese-Adjuvanted RBD-Fc Protein Induced the Highest Titers of nAbs against SARS-CoV-2 and Delta Variant

**Figure 4:**
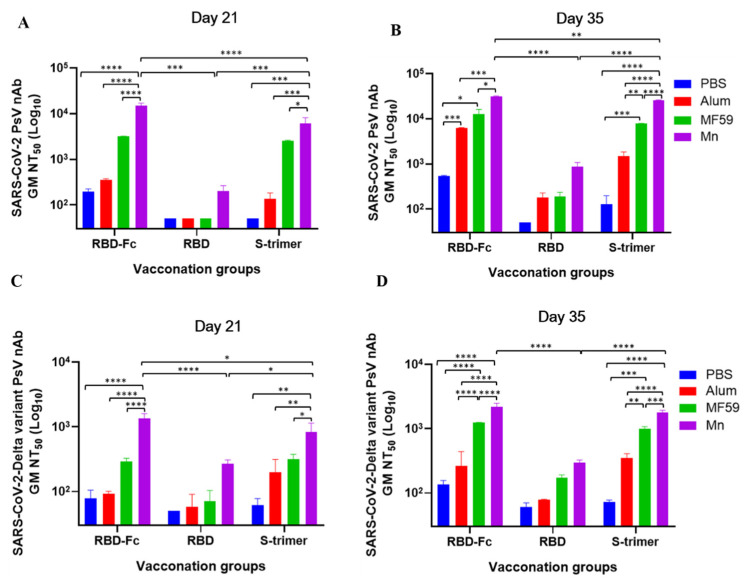
Neutralization activity observed in vaccinated mouse sera on days 21 and 35 against pseudotyped SARS-CoV-2 wildtype (D614G) and Delta variant. Mice immunized with different formulations were capable of producing neutralizing antibody responses to varying extents. Sera collected from mice on days 21 and 35 were evaluated for their content of neutralization antibodies. (**A**) nAbs against SARS-CoV-2 wildtype (D614G) pseudovirus by mouse sera collected on day 21. (**B**) nAbs against SARS-CoV-2 wildtype (D614G) pseudovirus by mouse sera collected on day 35. (**C**) nAbs against SARS-CoV-2 Delta variant pseudovirus by mouse sera collected on day 21. (**D**) nAbs against SARS-CoV-2 Delta variant pseudovirus by mouse sera collected on day 35. Statistical significance was determined by two-way ANOVA and is indicated as follows: * *p* < 0.05, ** *p* < 0.01, *** *p* < 0.001, **** *p* < 0.0001. Error bars represent SD.

We next detected the nAbs of all immunized mouse sera collected on days 21 and 35 against pseudotyped SARS-CoV-2 and its Delta variant. Compared with RBD and S-trimer, we found that RBD-Fc induced significantly higher nAbs against pseudotyped SARS-CoV-2 and that nanoparticle manganese was the most potent enhancer (average titer of 14,914 on days 21 and 31, 619 on day 35) (Figure 4A,B). As shown in Figure 4C,D, the vaccines also induced pseudotyped SARS-CoV-2 Delta-variant-specific nAbs. Here, nanoparticle-manganese-adjuvanted RBD-Fc also exhibited the most potent immunogen. However, compared with pseudotyped SARS-CoV-2, nAbs were significantly decreased (average titer of 1351 on day 21 and 2190 on day 35).

### 3.4. Manganese-Adjuvanted RBD-Fc Protein Effectively Reduced the Viral Load of the Authentic SARS-CoV-2 and Delta Variant in the Cell Culture Supernatants

**Figure 5:**
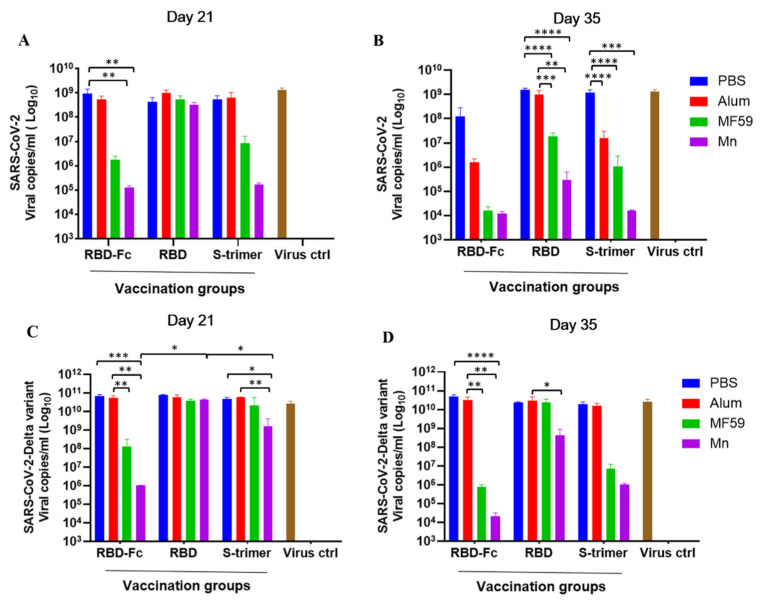
Viral load reduction assay in vaccinated mouse sera on days 21 and 35 against both the authentic SARS-CoV-2 wildtype (HKU-001a strain) and Delta variant. VeroE6-TMPRSS2 cells were used for viral infection, and the virus copies in the cell culture supernatants were quantified by real-time one-step (qRT-PCR). (**A**) Viral load reduction assay on SARS-CoV-2 wildtype (HKU-001a strain) by mouse sera collected on day 21. (**B**) Viral load reduction assay on SARS-CoV-2 wild-type (HKU-001a strain) by mouse sera collected on day 35. (**C**) Viral load reduction assay on SARS-CoV-2 Delta variant by mouse sera collected on day 21. (**D**) Viral load reduction assay on SARS-CoV-2 Delta variant by mouse sera collected on day 35. Statistical significance was determined by two-way ANOVA and is indicated as follows: * *p* < 0.05, ** *p* < 0.01, *** *p* < 0.001, **** *p* < 0.0001. Error bars represent SD.

We further detected the antiviral effect of all immunized mouse sera collected on days 21 and 35 against the authentic SARS-CoV-2 wildtype (HKU-001a strain) and Delta variant by using VeroE6-TMPRSS2 cells. Compared with RBD and S-trimer, we found that RBD-Fc effectively reduced the viral load of the authentic SARS-CoV-2 wildtype and Delta variant in cell culture supernatants on day 21, and nanoparticle manganese was the most potent enhancer (Figure 5A,C). As shown in Figure 5B,D, the mouse sera collected on day 35 obviously reduced the viral load in the supernatants, and nanoparticle manganese was the most potent enhancer.

## 4. Discussion

Infection with SARS-CoV-2 leads to COVID-19, the severity of which is partly due to host immune responses, including the release of proinflammatory cytokines, such as interleukin (IL)-6, IL-18 and tumor necrosis factor (TNF), with serious biological and clinical consequences [21,22]. Vaccination is considered one of the most effective strategies to fight against infectious diseases. However, vaccines with the potential to cause anaphylaxis should undergo strict safety and efficacy evaluations [23]. Adjuvants are commonly formulated in vaccines, and they have been demonstrated to enhance the immunogenicity and effectiveness of vaccine candidates [24].

Compared with alum, mPLA-SM, ISA51 and Freund’s adjuvants, we previously showed that MF59-adjuvanted S377-588-Fc was the most potent in inducing anti-MERS-CoV antibodies with neutralizing activity, as observed by the rapid virus clearance in mice after challenge with MERS-CoV [14]. Zhang R et al. showed that manganese-adjuvanted vaccines can stimulate strong humoral and cellular immune responses via either intramuscular (IM) or intranasal (IN) immunization by facilitating antigen uptake, presentation and germinal center formation through both cGAS-STING and NLRP3 activation [12]. In this study, we compared the enhancement of immune response among alum, MF59 and nanoparticle manganese via IM route. Among the three adjuvants evaluated, nanoparticle manganese, MF59 and Alum represent the most, the second most and the least potent enhancer of nAbs production in immunized mice, respectively. The detailed action mechanism of nanoparticle manganese adjuvant via IM route and animal challenge experiments will be further explored in the near future. In summary, a manganese-adjuvanted RBD-Fc vaccine candidate has the potential to induce extremely potent cross-protective nAbs against SARS-CoV-2 and its variants. Given the relatively low neutralizing immunogenicity, lack of broad-spectrum efficacy and minimal lasting protective immunity of current subunit vaccines, this adjuvanted RBD-Fc is promising for development as a next-generation COVID-19 candidate vaccine.

## Data Availability

All data are available from the corresponding authors upon reasonable request.
